# Expanding Research on Contextual Factors in Autism Research: What Took Us So Long?

**DOI:** 10.1002/aur.3312

**Published:** 2025-02-04

**Authors:** Marsha Mailick, Teresa Bennett, Leann Smith DaWalt, Maureen S. Durkin, Gordon Forbes, Patricia Howlin, Catherine Lord, Anat Zaidman‐Zait, Lonnie Zwaigenbaum, Vanessa Bal, Somer Bishop, Chung‐Hsin Chiang, Adriana DiMartino, Christine M. Freitag, Stelios Georgiades, Matthew Hollocks, Meng‐Chuan Lai, Matthew J. Maenner, Patrick S. Powell, Julie Lounds Taylor, Alycia Halladay

**Affiliations:** ^1^ Waisman Center University of Wisconsin Madison Wisconsin USA; ^2^ HHS/McMaster Children's Hospital McMaster University Hamilton Canada; ^3^ Kings College Institute of Psychiatry London UK; ^4^ University of California at Los Angeles Los Angeles California USA; ^5^ Tel‐Aviv University Tel‐Aviv Israel; ^6^ University of Alberta Edmonton Canada; ^7^ Autism Research Centre Glenrose Rehabilitation Hospital Edmonton Canada; ^8^ Rutgers University–New Brunswick New Brunswick New Jersey USA; ^9^ Rutgers Center for Adult Autism Services New Brunswick New Brunswick New Jersey USA; ^10^ University of California at San Francisco San Francisco California USA; ^11^ National Chengchi University Taipei Taipei City Taiwan; ^12^ Child Mind Institute New York New York USA; ^13^ University Hospital Frankfurt Goethe‐Universität Frankfurt Germany; ^14^ Center for Addition and Mental Health University of Toronto Toronto Ontario Canada; ^15^ Department of Psychiatry University of Cambridge Cambridge UK; ^16^ Centers for Disease Control and Prevention National Center on Birth Defects and Developmental Disabilities Atlanta Georgia USA; ^17^ Vanderbilt University Medical Center Nashville Nashville Tennessee USA; ^18^ Autism Science Foundation New York New York USA; ^19^ Rutgers University Department of Pharmacology and Toxicology Piscataway New Jersey USA

**Keywords:** autism, behavior, contextual, longitudinal, outcomes, research

## Abstract

Although autism is a childhood‐onset neurodevelopmental disorder, its features change across the life course due to a combination of individual and contextual influences. However, the influence of contextual factors on development during childhood and beyond is less frequently studied than individual factors such as genetic variants that increase autism risk, IQ, language, and autistic features. Potentially important contexts include the family environment and socioeconomic status, social networks, school, work, services, neighborhood characteristics, environmental events, and sociocultural factors. Here, we articulate the benefit of studying contextual factors, and we offer selected examples of published longitudinal autism studies that have focused on how individuals develop within context. Expanding the autism research agenda to include the broader context in which autism emerges and changes across the life course can enhance understanding of how contexts influence the heterogeneity of autism, support strengths and resilience, or amplify disabilities. We describe challenges and opportunities for future research on contextual influences and provide a list of digital resources that can be integrated into autism data sets. It is important to conceptualize contextual influences on autism development as main exposures, not only as descriptive variables or factors needing statistical control.

**Summary**

- Autism is known to be affected by a variety of contextual factors across the life course, including the family environment and socioeconomic status, social networks, school, work, services, neighborhood characteristics, environmental events, and sociocultural factors.
- These factors are rarely studied by autism researchers but can have substantial influences on the outcomes of autistic children and adults.
- In the paper, we encourage researchers to pay more attention to these factors and describe challenges and opportunities for future research.

**Abbreviations**
ASD: autism spectrum disorder
GIS: geographic information system
IQ: intelligence quotient
SES: socioeconomic status

## 1 Introduction

Contextual factors are aspects of the environment that affect the development and functioning of a person and can include social, cultural, economic, political, physical, and other such influences. Although there is broad recognition of the importance of contextual factors in the larger research literature on child and adult development, this perspective has been less prominent in autism research. Rather, autism studies have largely been focused on individual and biological factors that can affect development, such as genetic variants that increase autism risk, gender, and cognitive and language abilities, among others. In contrast, more limited research has been conducted on how contextual factors that are external to the individual contribute to individual variation and change from childhood to adulthood.

Contextual factors can be conceptualized along a continuum, whereby the most proximal factors (e.g., the family) are those most closely surrounding the autistic individual, while the most distal factors (e.g., the neighborhood and physical environment) are independent of the individual. Research designs in autism can be strengthened by including a focus on how contextual factors along this continuum contribute to individual development and outcomes. Investigation of contextual factors, together with individual and biological variables, has the potential to enrich understanding of the heterogeneity in the developmental course of autism.

In 2023, the Autism Science Foundation invited a group of autism researchers and clinicians to consider the importance of contextual factors in autism research and to offer suggestions for expanding the inclusion of contextual factors in such studies. The group included 21 scientists who met monthly over the course of a year, discussed the most prominent challenges in incorporating contextual factors in autism research, and collaborated on this commentary. There was recognition of the importance of including researchers from both Western and non‐Western countries who have conducted longitudinal autism research; yet most such research has been conducted in Western countries, and thus most of the co‐authors of the present manuscript reflect this limitation. Here, we seek to draw attention to the importance of contextual factors in autism research, provide selected examples of how such factors already have been incorporated in studies of autism, and enumerate digital resources for researchers who would be interested in including these factors in their own research. In addition, we offer a list of Supporting Information references that might be valuable to researchers in future studies seeking to incorporate contextual factors in autism research.

### 1.1 Why Focus on Contextual Factors in Longitudinal Autism Research?

We focus here on *longitudinal* research, recognizing that consideration of contextual factors can enhance multiple approaches to the study of autism, including cross‐sectional designs. However, longitudinal autism studies are best positioned to reveal how contexts shape individual development over time. Not all contextual influences are evident at a single time; some have lagged effects or result from cumulative processes, only over time reaching a threshold when linkages between prior exposure to contexts and individual outcomes become apparent (see (Lai 2023) and McKinney (McKinney et al. 2024) for examples). Additionally, there are periods during development when contextual factors have a disproportionate impact on autistic children and adults, such as exposure to adversity during the first years of life (Hartley et al. 2023) or exit from high school (Clarke, McCauley, and Lord 2021; Taylor and Seltzer 2011). Importantly, contexts themselves are not static. Contexts such as family, neighborhood, and societal factors also change over time, and longitudinal research offers the best opportunity to directly examine change in contexts and how such changes can offer greater or more constrained opportunities for individual growth.

### 1.2 Age‐Period‐Cohort Effects

Autism studies are subject to age, period, and cohort effects, but few are guided by this paradigm. *Age effects*, a central focus of autism research, include variations in symptoms, health, and functioning that are associated with age‐related physiological or psychological development. In contrast, *period effects* reflect contextual factors. These include events that occur at particular periods of time such as economic disruptions, pandemics, war, and famine, as well as to policy or definitional changes that affect who is identified as autistic or who is eligible for services. *Cohort effects* result from the sum of experiences shared by individuals in a birth cohort or other temporally defined cohort and may be seen as interactions between age and period effects (Yang and Land 2013).

Contextual factors can help explain whether sub‐groups of the autistic population are differentially affected by period effects. For example, the economic recession of 2007–2009 affected access to autism diagnostic and treatment services, with potential delays in diagnosis that resulted in increases in mental health challenges for autistic individuals and their parents (Hickey et al. 2024). However, without inclusion of measures of parental educational attainment and income in studies of the impact of the recession, it would not be possible to determine the extent to which the impact of the recession on autism diagnosis varied by socioeconomic status. Similarly, contextual factors could explain whether the COVID‐19 pandemic (Eshraghi et al. 2022; Lee et al. 2021; Pokoski, Crain, DiGuiseppi, et al. 2024; Pokoski, Crain, Furnier, et al. 2024; Vibert et al. 2023) differentially impacted the behavior and development of some autistic children more than others, depending on the availability of health care and testing sites in their neighborhoods. Recognition of cohort effects, and their inclusion in longitudinal autism research, makes it possible to evaluate how the ever‐changing social, economic, and policy landscape impacts how autistic individuals develop and adapt.

### 1.3 Selection of Contextual Factors in Autism Research

Selection of contextual variables for a study depends on many factors: the research question being addressed, the stage of life of the autistic individuals, the heterogeneity of the participants being studied, and the range of outcomes that are investigated. For example, studies of health of autistic people could be enhanced by inclusion of measures of neighborhood characteristics, systemic discrimination, social attitudes, ability to access health and social services, social support networks, or poverty, all of which can profoundly influence the health of autistic individuals over time (Stringer et al. 2020). These factors are associated with health disparities, which, according to the U.S. Centers for Disease Control and Prevention, are preventable differences in the burden of disease or in opportunities to achieve optimal health (US Centers for Disease Control and Prevention 2023). Without such contextual measures, important sources of variance in health outcomes among autistic people cannot be accounted for and might be incorrectly assumed to be inherent to autism.

Studies of service utilization by autistic people can be enhanced by inclusion of measures of differential opportunities in society, such as race and ethnicity, to clarify the extent to which sub‐groups have differential access to services. However, a recent systematic review of primarily US studies found that only 25% of published autism intervention studies reported any data on race and ethnicity (Steinbrenner et al. 2022), even though the importance of race and ethnicity have been well established in many other fields and even though reporting of race and ethnicity is required by the U.S. (National Institutes of Health n.d.; National Institutes of Health S.1103rd Congress (1993–1994) n.d.) and increasingly by many journals. The importance of contextual factors such as socioeconomic status is evident in non‐Western societies as well as in Western. For example, in Taiwan, ongoing longitudinal follow‐up studies of autistic children (diagnosed in preschool years) illustrate the complex impact of services and resources in the context of a high‐income, non‐Western country (Chu et al. 2017; Liu 2022). Specifically, such studies demonstrate that family socio‐economic status and service resources interact to differentially influence the developmental outcomes of autistic children and those with other developmental disabilities, evidencing the complex impact of services and resources in the context of a high‐income, non‐Western country.

Contextual factors separately and synergistically have been shown to influence typical development and warrant greater inclusion in autism research. Although no single study can reveal all of these complex processes, the inclusion of relevant contextual factors in multiple studies can cumulatively build an understanding of their effects in autism research.

### 1.4 Questions, Challenges, and Opportunities

Here, we identify just a few questions, challenges, and opportunities that warrant consideration in moving toward the inclusion of contextual factors in autism research. One important *question* is how best to statistically analyze change when there are so many moving parts—in contextual factors themselves, in other independent variables, and in dependent variables. Different types of questions need different statistical approaches. Examples include modeling discontinuities in development as a function of contextual factors, detecting how individual characteristics interact with contextual factors to influence development, and linking the dynamics of contextual factors to the dynamics of individual autism trajectories (Chen et al. 2023).

Among the *challenges* faced when including contextual factors in autism research is the diversity of ways to measure contextual factors, as they vary enormously across geopolitical lines and over time, making harmonization of data difficult. In addition, comparing cohorts from different historical periods may be problematic due to changes over time in diagnostic criteria, health care, services, and educational opportunities for autistic people, which influence outcomes. Research has also struggled with quantifying the types of interventions that change across the lifespan and determining their interactions with contextual factors. Addressing this need in future research could lead to more personalized, more effective interventions (Lord et al. 2022). Finally, memory and reporting of contextual factors by autistic adults and family members are challenging to contextual factor research.

*Opportunities* that merit consideration in future autism research include digital resources about specific geographic locations that are easily accessible on‐line and can significantly expand the range of contextual factors studied. Examples can be found in Table 1, such as neighborhood SES and Geographic Information System (GIS) databases for access to community resources. The digital resources in Table 1 were selected because of ease of access worldwide and because they are free of charge. Thus, they offer the opportunity for integration into existing autism datasets.

**TABLE 1:** List, Weblinks, descriptors, and citations of data resources that capture information on contextual factors in autism research.

Name of resource and link	Location	Description	Example
ArcGIS software	World	Geographic information systems to map proximity to identified features or number of features from a location	Chan et al. (2021)
Health Registry Data	World	Specifically in Norway, Denmark, Sweden, and some places in California, electronic health records paired with biological data	Persson et al. (2020)
Human Development Index	World	This is a summary of an average achievement in key dimensions like life expectancy, standard of living, and years of education.	Borumandnia et al. (2022)
NASA Goddard Earth Observing System	World	Coordinated a series of satellites to monitor land surface, biosphere, atmosphere, and oceans	McGuinn et al. (2021)
UNICEF	World	World statistics on a variety of factors including infant and child development, education, migration, mortality, and child protection.	UNICEF (2021)
American Community Survey	US	Ongoing community survey that evaluates jobs, occupations, educational attainment, language used, benefits recieved, living expenses, and income. Data are shared on a neighborhood level.	Dunlop et al. (2023)
Area Deprivation Index	US	Measures of neighborhood disadvantage using income, education, employment, and housing quality.	Magana et al. (2023)
California Department of Developmental Services	US	To inform policy and enhance oversight, DDS collects data from many different parts of the service delivery system, including individuals and families.	Fountain et al. (2023)
CDC/ATSDR Social Vulnerability Index	US	Uses 16 US census variables to help local officials identify social vulnerability that may need support.	Durkin et al. (2017); Patrick et al. (2023)
Child Opportunity Index	US	Measures and maps the quality of resources and conditions such as schools, parks, playgrounds, air quality, food quality, health care, and housing.	Fisher and Lynch (2024)
Environmental Protection Agency Emissions‐Based National‐Scale Air Toxics Assessment	US	National screening assessment that uses emissions and weather data to estimate health risks from toxic air pollutants.	Dickerson et al. (2016); Kalkbrenner et al. (2018)
Socio‐Economic Indexes for Areas	Australia	Ranks areas according to their relative socio‐economic advantage and disadvantage using health data.	Abdullahi et al. (2023)
Canadian Taxfilers	Canada	Annual information on Canadians who filed taxes: number, age, median income, and employment income by postal codes.	Forer et al. (2019); Guhn et al. (2016); Siddiqua, et al. (2020).
Tipat Halav: Maternal Child Health Clinics	Israel	Free health clinics that provide preventative care and collect data on child health problems, immunizations, and developmental milestones.	Drotar et al. (2008)

Methods developed for causal inference (e.g., natural experiments) present an *opportunity* to move beyond describing associations to understanding causal mechanisms (De Stavola, Herle, and Pickles 2022). An outcome‐wide approach, examining the effect of a single contextual factor across many facets of life for an autistic person, can simplify the identification of causal effects and deliver better public health recommendations (VanderWeele 2017). These factors could be better understood through research that incorporates hypothesized mediation pathways linking more distal to proximal contextual influences. Furthermore, incorporating a stronger understanding of moderation of contextual effects can inform “what applies and for whom” with respect to risk and protection across heterogeneous profiles of autistic children and adults.

Finally, the dynamic nature of contextual factors presents both a *challenge and an opportunity*. Changes in context can be measured repeatedly over time and evaluated in conjunction with intra‐individual change. Additionally, identification of influential contextual factors offers *opportunities* for designing programmatic and policy interventions. However, when contextual variables change (such as changes in the cut‐off of the “poverty line”), there are *challenges* in how to harmonize repeated measures over time, as well as the *opportunity* to explore the dynamic nature of context.

### 1.5 Future Directions

Currently, there is a paucity of research that conceptualizes contextual influences on autism development as *main exposure variables*, not only as descriptive variables or factors needing statistical control. Existing scoping reviews about contextual factors (Ng et al. 2017); (Anderson et al. 2018); (Greenlee, Winter, and Diehl 2018) may be a place to start addressing this gap, although these reviews have tended to focus on specific contextual factors during select time periods and on various sub‐sets of the autism population. Nevertheless, autism researchers could use these reviews as resources when planning to incorporate contextual factors into longitudinal studies.

A consequential next step could be to turn to the literature beyond autism and consider contextual factors that have been shown to affect lifespan development in the broader population. General frameworks from public health, epidemiology, sociology, and disability studies (e.g., the World Health Organization's International Classification of Disability, Black et al. 2023) could be used for this purpose. As the social model of disability (Woods 2017) calls attention to person‐environment fit, knowledge about how contextual factors can enhance development can move the autism field toward broader strategies for improving outcomes beyond focus on individual characteristics as predictors or targets of intervention.

In addition, research findings from the general population on the effects of contextual factors may guide future autism research. For example, a recent meta‐analysis (603 studies from 59 countries) in *Lancet Public Health* reported that years of schooling showed a dose–response relationship with adult mortality—a 1.9% reduction in mortality risk for each additional year of education (IHME‐CHAIN Collaborators 2024). These results have potentially important implications for the effects of education on health and mortality in autistic populations.

Contextual factors can be malleable, potentially improving the lives of autistic individuals (Dawson 2008; Pickles et al. 2016). Knowledge from research that incorporates measures of contextual factors could be used to build contexts that support positive outcomes for autistic people, including those with and without intellectual disability (Shogren, Luckasson, and Schalock 2018). This knowledge could influence advocacy efforts (e.g., for access to higher education), the design of services (e.g., support for families), and evaluation of interventions. Furthermore, expanding international collaborations focused on contextual factors in autism longitudinal research and developing funding mechanisms that can support the study of the impact of larger contextual factors across the world remains a crucial next step in broadening the field's understanding of how the context influences individual development.

In conclusion, the study of the impact of contextual factors opens the possibility for expanding understanding of the heterogeneity of autism, improving representativeness in research, and answering important questions about lifespan development that have thus far eluded the field, thus enhancing the relevance of future autism research. As autism researchers, we ask the question of our field—what took us so long?

## Ethics Statement

The authors have nothing to report.

## Conflicts of Interest

The authors declare no conflicts of interest.

## Supporting information


Data S1.

## Data Availability

Data sharing not applicable to this article as no datasets were generated or analysed during the current study.
