# Supplementary material for: Expanding Research on Contextual Factors in Autism Research: What Took Us So Long?
Source: Autism Res. 2025 Feb 4;18(4):710–6. doi: 10.1002/aur.3312 (PMC12015804; doi:10.1002/aur.3312)
Supplement: Supplementary file 1 — Data S1. [file AUR-18-710-s001.docx]

**Supplement 1: Full Reference List**

Bowleg, L. (2012). The Problem With the Phrase *Women and Minorities:* Intersectionality—An Important Theoretical Framework for Public Health. *American Journal of Public Health*, *102*(7), 1267–1273. <https://doi.org/10.2105/AJPH.2012.300750>

Caplan, B., Blacher, J., Eisenhower, A., Baker, B. L., & Lee, S. S. (2021). Gene x responsive parenting interactions in social development: Characterizing heterogeneity in autism spectrum disorder. *Developmental Psychobiology*, *63*(5), 1082–1097. <https://doi.org/10.1002/dev.22095>

Carr, T., & Lord, C. (2013). Longitudinal study of perceived negative impact in African American and Caucasian mothers of children with autism spectrum disorder. *Autism*, *17*(4), 405–417. <https://doi.org/10.1177/1362361311435155>

Del Bianco, T., Lockwood Estrin, G., Tillmann, J., Oakley, B. F., Crawley, D., San José Cáceres, A., Hayward, H., Potter, M., Mackay, W., Smit, P., Du Plessis, C., Brink, L., Springer, P., Odendaal, H., Charman, T., Banaschewski, T., Baron-Cohen, S., Bölte, S., Johnson, M., … the EU-AIMS LEAP Team. (2024). Mapping the link between socio-economic factors, autistic traits and mental health across different settings. *Autism*, *28*(5), 1280–1296. <https://doi.org/10.1177/13623613231200297>

Elias, R., & Lord, C. (2022). Diagnostic stability in individuals with autism spectrum disorder: Insights from a longitudinal follow‐up study. *Journal of Child Psychology and Psychiatry*, *63*(9), 973–983. <https://doi.org/10.1111/jcpp.13551>

Hickey, E. J., Bolt, D., Rodriguez, G., & Hartley, S. L. (2020). Bidirectional Relations between Parent Warmth and Criticism and the Symptoms and Behavior Problems of Children with Autism. *Journal of Abnormal Child Psychology*, *48*(6), 865–879. <https://doi.org/10.1007/s10802-020-00628-5>

Hume, K., Odom, S. L., Steinbrenner, J. R., Smith DaWalt, L., Hall, L. J., Kraemer, B., Tomaszewski, B., Brum, C., Szidon, K., & Bolt, D. M. (2022). Efficacy of a School-Based Comprehensive Intervention Program for Adolescents With Autism. *Exceptional Children*, *88*(2), 223–240. <https://doi.org/10.1177/00144029211062589>

King, M., & Bearman, P. (2009). Diagnostic change and the increased prevalence of autism. *International Journal of Epidemiology*, *38*(5), 1224–1234. <https://doi.org/10.1093/ije/dyp261>

Lai, M.-C., Anagnostou, E., Wiznitzer, M., Allison, C., & Baron-Cohen, S. (2020). Evidence-based support for autistic people across the lifespan: Maximising potential, minimising barriers, and optimising the person–environment fit. *The Lancet Neurology*, *19*(5), 434–451. <https://doi.org/10.1016/S1474-4422(20)30034-X>

Lord, C., Charman, T., Havdahl, A., Carbone, P., Anagnostou, E., Boyd, B., Carr, T., De Vries, P. J., Dissanayake, C., Divan, G., Freitag, C. M., Gotelli, M. M., Kasari, C., Knapp, M., Mundy, P., Plank, A., Scahill, L., Servili, C., Shattuck, P., … McCauley, J. B. (2022). The Lancet Commission on the future of care and clinical research in autism. *The Lancet*, *399*(10321), 271–334. <https://doi.org/10.1016/S0140-6736(21)01541-5>

Lord, C., McCauley, J. B., Pepa, L. A., Huerta, M., & Pickles, A. (2020). Work, living, and the pursuit of happiness: Vocational and psychosocial outcomes for young adults with autism. *Autism*, *24*(7), 1691–1703. <https://doi.org/10.1177/1362361320919246>

Maltman, N., DaWalt, L. S., Hong, J., & Mailick, M. (2021). Brief Report: Socioeconomic Factors Associated with Minimally Verbal Status in Individuals with ASD. *Journal of Autism and Developmental Disorders*, *51*(6), 2139–2145. <https://doi.org/10.1007/s10803-020-04646-6>

Pickles, A., McCauley, J. B., Pepa, L. A., Huerta, M., & Lord, C. (2020). The adult outcome of children referred for autism: Typology and prediction from childhood. *Journal of Child Psychology and Psychiatry*, *61*(7), 760–767. <https://doi.org/10.1111/jcpp.13180>

Piven, J., Harper, J., Palmer, P., & Arndt, S. (1996). Course of Behavioral Change in Autism: A Retrospective Study of High-IQ Adolescents and Adults. *Journal of the American Academy of Child & Adolescent Psychiatry*, *35*(4), 523–529. <https://doi.org/10.1097/00004583-199604000-00019>

Radler, B. T., & Ryff, C. D. (2010). Who Participates? Accounting for Longitudinal Retention in the MIDUS National Study of Health and Well-Being. *Journal of Aging and Health*, *22*(3), 307–331. <https://doi.org/10.1177/0898264309358617>

Smith, J., Chetcuti, L., Kennedy, L., Varcin, K. J., Slonims, V., Bent, C. A., Green, J., Iacono, T., Pillar, S., Taylor, C., Wan, M. W., Whitehouse, A. J. O., Hudry, K., & the AICES Team. (2023). Caregiver sensitivity predicts infant language use, and infant language complexity predicts caregiver language complexity, in the context of possible emerging autism. *Autism Research*, *16*(4), 745–756. <https://doi.org/10.1002/aur.2879>

Song, J., Dembo, R. S., Smith DaWalt, L., Ryff, C. D., & Mailick, M. R. (2023). Improving Retention of Diverse Samples in Longitudinal Research on Developmental Disabilities. *American Journal on Intellectual and Developmental Disabilities*, *128*(2), 164–175. <https://doi.org/10.1352/1944-7558-128.2.164>

Sturm, A., Williams, J., & Kasari, C. (2021). Who gains and who loses? Sociodemographic disparities in access to special education services among autistic students. *Autism Research*, *14*(8), 1621–1632. <https://doi.org/10.1002/aur.2517>

Taylor, J. L., Smith, L. E., & Mailick, M. R. (2014). Engagement in Vocational Activities Promotes Behavioral Development for Adults with Autism Spectrum Disorders. *Journal of Autism and Developmental Disorders*, *44*(6), 1447–1460. <https://doi.org/10.1007/s10803-013-2010-9>

Tesfaye, R., Courchesne, V., Mirenda, P., Mitchell, W., Nicholas, D., Singh, I., Zwaigenbaum, L., & Elsabbagh, M. (2023). Autism voices: Perspectives of the needs, challenges, and hopes for the future of autistic youth. *Autism*, *27*(4), 1142–1156. <https://doi.org/10.1177/13623613221132108>

West, E. A., Travers, J. C., Kemper, T. D., Liberty, L. M., Cote, D. L., McCollow, M. M., & Stansberry Brusnahan, L. L. (2016). Racial and Ethnic Diversity of Participants in Research Supporting Evidence-Based Practices for Learners With Autism Spectrum Disorder. *The Journal of Special Education*, *50*(3), 151–163. <https://doi.org/10.1177/0022466916632495>

Winter, A. S., Fountain, C., Cheslack-Postava, K., & Bearman, P. S. (2020). The social patterning of autism diagnoses reversed in California between 1992 and 2018. *Proceedings of the National Academy of Sciences*, *117*(48), 30295–30302. <https://doi.org/10.1073/pnas.2015762117>

Woodman, A. C., Smith, L. E., Greenberg, J. S., & Mailick, M. R. (2015). Change in Autism Symptoms and Maladaptive Behaviors in Adolescence and Adulthood: The Role of Positive Family Processes. *Journal of Autism and Developmental Disorders*, *45*(1), 111–126. <https://doi.org/10.1007/s10803-014-2199-2>

Zaidman-Zait, A., Mirenda, P., Duku, E., Vaillancourt, T., Smith, I. M., Szatmari, P., Bryson, S., Fombonne, E., Volden, J., Waddell, C., Zwaigenbaum, L., Georgiades, S., Bennett, T., Elsabaggh, M., & Thompson, A. (2017). Impact of personal and social resources on parenting stress in mothers of children with autism spectrum disorder. *Autism*, *21*(2), 155–166. <https://doi.org/10.1177/1362361316633033>
